# Early Experience Using Tantalum-Loaded Nanocomposite Hydrogel Conformable Embolic for Upper Gastrointestinal Bleeding-Open-Sandwich Technique

**DOI:** 10.3390/jcm14072345

**Published:** 2025-03-29

**Authors:** Sandra Gad, Lourens Du Pisanie, Michael Mohnasky, Bryan Harris, Alexander Villalobos, Nicole Keefe, Priya Mody, Andrew Caddell, Nima Kokabi

**Affiliations:** 1 Division of Vascular & Interventional Radiology, Department of Radiology, University of North Carolina at Chapel Hill, Chapel Hill, NC 27599, USA; 2 School of Medicine, St. George’s University, St. George’s, West Indes P.O. Box 7, Grenada; 3 School of Medicine, University of North Carolina at Chapel Hill, Chapel Hill, NC 27599, USA

**Keywords:** upper gastrointestinal bleeding, embolization, conformable embolic, gastroduodenal artery

## Abstract

**Background/Objectives**: To evaluate the efficacy and safety of using tantalum-loaded Obsidio conformable embolic (Ta-OCE) in gastroduodenal artery (GDA) embolization for upper gastrointestinal bleeding (UGIB), employing a novel “open-sandwich” technique. **Methods**: An institutional review board (IRB)-approved retrospective analysis was conducted on patients who underwent GDA embolization for UGIB using Ta-OCE between May 2023 and June 2024, using an “open-sandwich” technique. Briefly, the retrograde sources of flow, namely the right gastroepiploic artery (RGEA), was commonly embolized with a single, usually detachable, coil at its proximal aspect. Beginning within the proximal RGEA adjacent to the coil and distal to the site of extravasation and/or an endoscopically placed clip, Ta-OCE was then instilled in a continuous fashion to the origin of GDA. Technical success was defined as complete occlusion of the target vessel without immediate procedural complications. Clinical success was assessed as the absence of rebleeding within 4 weeks post-embolization. Adverse events were evaluated using Common Toxicity Criteria for Adverse Events (v.5). **Results**: Overall, a total of 10 patients, with a mean age of 67.3 years, underwent Ta-OCE embolization for UGIB. A technical success rate of 100% was achieved with no instances of immediate procedural complications. Clinical success was achieved in eight patients (80%). Re-intervention was required in two patients in whom the proximal GDA and distal GDA/proximal RGEA were not embolized adequately, respectively. A significant change in mean hemoglobin levels was observed 24 h pre- and post-embolization, with a mean increase of 1.47 g/dL. **Conclusions**: Despite the small sample size, lack of control group, and retrospective design, the “open-sandwich” technique combining Ta-OCE with a single coil appears to be an effective and safe method of GDA embolization in the setting of UGIB. Larger multicenter studies are needed to further evaluate the feasibility of this technique.

## 1. Introduction

Upper gastrointestinal bleeding (UGIB) is a common and potentially deadly medical condition with an estimated annual incidence of 80–150 cases per 100,000 individuals [1,2]. Its associated with an estimated mortality rate of 10%. Several etiologies cause UGIB, including gastric malignancies, esophagitis, and vascular abnormalities, with peptic ulcers being the most common cause [3,4]. In managing UGIB, prompt endoscopic intervention after initial resuscitation is established as a first-line approach to achieve hemostasis [5,6]. Endoscopy offers both diagnostic and therapeutic benefits, allowing direct visualization of the bleeding source and application of hemoclips or contact thermocoagulation [6]. However, in 10–20% of patients, endoscopic therapy may not be feasible due to anatomical challenges, or may fail due to active rebleeding or the presence of large, complex ulcers [7]. Transcatheter arterial embolization (TAE) has emerged as the preferred secondary therapeutic strategy when patients have failed endoscopic and conservative medical therapies [3,8,9]. In fact, TAE has surpassed surgery as the treatment of choice for endoscopy-refractory UGIB [9,10].

Whether prophylactic or for active arterial contrast extravasation, the gastroduodenal artery (GDA) is a frequent target for embolization in UGIB that has failed endoscopic therapy [11,12]. Various embolic agent options are available for GDA embolization, including absorbable gelatin sponge (gel foam), plugs, and coils. Liquid agents, including N-butyl 2-cyanoacrylate (NBCA) and ethylene-vinyl alcohol copolymer (Onyx^®^, Micro Therapeutics, Inc., Irvine, CA, USA), could also be considered in certain cases [13,14]. Each embolic has unique properties that influence its use; factors such as vessel size, the presence of collaterals, preparation time, the patient’s coagulopathic status, and operator expertise are the most common factors that tend to play a role in their utility [15].

Among the described techniques, the “sandwich technique” is a well-established method for GDA embolization [8,12]. This technique involves embolizing retrograde sources of blood flow (e.g., the right gastroepiploic and pancreaticoduodenal arteries) with coils or plugs, followed by gel foam slurry instillation within the GDA and, finally, embolization of the main trunk using a coil [8,12]. This technique has been shown to have good technical and clinical success rates ranging from 69 to 100% and from 63 to 97%, respectively [16,17].

Obsidio^®^ (Boston Scientific, Marlborough, MA, USA) is a novel tantalum-loaded nanocomposite hydrogel conformable embolic that conforms to the target vessel’s shape and provides mechanical occlusion [18]. The tantalum-loaded Obsidio conformable embolic (Ta-OCE) is FDA 510k cleared for embolization of hypervascular tumors and bleeding in peripheral vessels with diameters ≤3 mm [19,20,21].

Previous studies using swine models suggest a number of potential benefits, such as ease of delivery and minimal off-target embolization, while capitalizing on the advantageous characteristics of the liquid embolic [22]. However, there is a paucity of clinical data regarding its safety and efficacy in several clinically relevant scenarios [19,20]. This study aims to evaluate the technical success, short-term safety, and effectiveness of Ta-OCE embolization of the GDA in UGI bleeds, using an augmented two-step approach to the sandwich technique for GDA embolization, coined the “open-sandwich” technique, which utilizes this conformable embolic in conjunction with a single coil in the “backdoor” of the GDA.

Furthermore, this study aims to provide insights into the benefits and challenges of incorporating this novel embolic material into clinical practice in the setting of UGIB.

## 2. Materials and Methods

### 2.1. Data Collection

**Figure 1:**
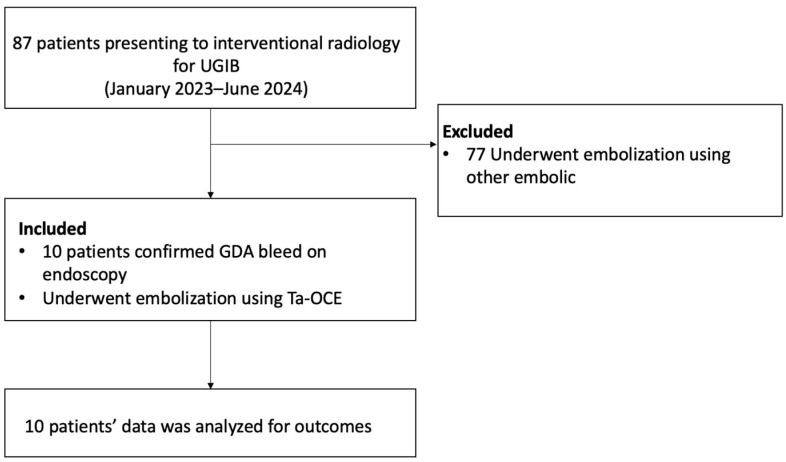
A flow diagram of patient inclusion and exclusion.

This is a retrospective Health Insurance Portability and Accountability Act (HIPAA)-compliant study, which was approved by the Institutional Review Board (IRB). Patients were excluded if a different embolic agent was used. All patients who underwent trans-arterial embolization procedures using Ta-OCE for GDA bleeding confirmed by endoscopy were included in this retrospective observational study (Figure 1). Data were collected retrospectively for consecutive patients who underwent transcatheter angiography for GDA embolization with Ta-OCE from January 2023 to June 2024.

### 2.2. Procedure Details

**Figure 2:**
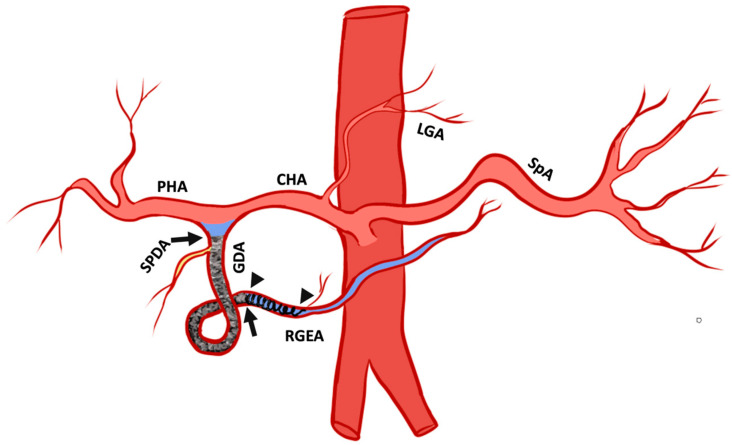
Pictogram of the open-sandwich technique: The arrowheads mark the beginning and end of a single coil in the right gastroepiploic artery RGEA. The arrows outline the beginning and end of the conformable embolic deployed within the proximal RGEA after the coil to the origin of the gastroduodenal artery (GDA) proximal to the take-off the superior pancreaticoduodenal artery (SPDA). Proper hepatic artery (PHA), common hepatic artery (CHA), left gastric artery (LGA), and splenic artery (SPA). The pictogram was developed by the authors.

The fundamental techniques used to embolize GDA have been previously reported [2]. We describe some technical variations based on the evolution of the authors’ experience, where the authors adapted the “sandwich technique” to capitalize on Ta-OCE’s sheer thinning embolic feature, which we termed the “Open-Sandwich technique” (Figure 2).

Board-certified interventional radiologists performed all the embolization procedures. Celiac axis angiography was performed using a 5 Fr base catheter to determine the arterial anatomy and active arterial extravasation. The most common 5 Fr catheters used were the Cobra 2 (Merit Medical, Salt Lake City, UT, USA) and Sos 1 (AngioDynamics, Latham, NY, USA) for transfemoral access and MG1 (Terumo Medical, Tokyo, Japan) for transradial. Subsequently, using a co-axial technique, a microcatheter was used to select the GDA. The most commonly used microcatheters were the 2.0 Fr Truselect (Boston Scientific, Marlbrough, MA, USA) and 2.4 Fr Progreat (Terumo Medical, Tokyo, Japan). The microcatheter was then advanced distal to the bleeding site using a microwire (Meister16, Asahi Intecc, Aichi, Japan). The potential retrograde sources of flow, namely the right gastroepiploic artery (RGEA), was commonly embolized with a single, usually detachable, coil at its proximal aspect. Beginning within the proximal RGEA adjacent to the coil and distal to the site of extravasation and or endoscopically placed clip, Ta-OCE was then instilled in a continuous fashion. This was continued to the origin of GDA. During this process, the Ta-OCE also embolized other inflows, including those from pancreaticoduodenal arcade (PDA). Care was taken to embolize past the most proximal branches of the GDA, namely the superior pancreaticoduodenal artery (SPDA). The microcatheter containing residual Ta-OCE was then removed. Confirmatory angiography was then performed through the base catheter. Technical success was defined as achieving complete stasis on angiography, indicated by a static contrast column for at least five consecutive heartbeats. The base catheter was then removed, and hemostasis was achieved with a closure device for transfemoral access and with a transradial band for the transradial approach. The choice of vascular access—transfemoral or transradial—was determined by operator preference and patient anatomy. Following the embolization, computed tomography angiogram (CTA) was obtained in 24–48 h to assess target vessel patency and presence of non-target radiopaque Ta-OCE and to survey for adverse events if warranted. Embolization was defined as technically successful when no contrast extravasation from the target vessel was seen and/or with complete occlusion of the target artery. Clinical success was defined as a lack of further bleeding for 30 days. We present a case-based pictorial review of various Ta-OCE embolization cases encountered at our institution.

### 2.3. Preintervention Clinical Characteristics

The following preintervention characteristics were extracted from medical records: age, sex, the underlying cause of bleeding, hemodynamic status, and indication for GDA embolization. The patients were followed up for 4 weeks and 6 months post-intervention.

### 2.4. Statistical Analyses

Continuous variables are expressed as mean and SD. Categoric variables are expressed as frequency and proportion. Mean hemoglobin levels were observed 24 h pre- and post-embolization and were compared using a paired *t*-test. All statistical analyses were performed using R software version 4.2.3 (R Foundation for Statistical Computing, Vienna, Austria).

## 3. Results

### 3.1. Clinical Characteristics

This retrospective observational study included ten (*n* = 10) patients with an average age of 67.3 ± 15.2 years. Among them, two (20%) were female. The underlying causes of bleeding varied. Duodenal or gastric ulcers accounted for half of the cases (50%, *n* = 5) (Table 1). At the time of embolization, 60% of patients were hemodynamically unstable. Empiric gastroduodenal artery (GDA) embolization was performed in six patients (60%), while four (40%) underwent embolization for active bleeding.

### 3.2. Technical and Clinical Efficacy

Seven patients underwent embolization via left or right common femoral arterial access, whereas three patients underwent embolization via left radial arterial access. The reasons for using Ta-OCE included an emergent need for rapid hemostasis and/or operator preference. A significant change in mean hemoglobin levels was observed 24 h pre- and post-embolization, with a mean increase of 1.47 g/dL (*p* = 0.0001). Since the detection of bleeding, each patient received a mean of 2.8 units of blood transfusions pre-embolization (range: 0–7 transfusions) and 1.6 units (range: 1–4 transfusions) post-embolization (*p* = 0.167).

**Figure 3:**
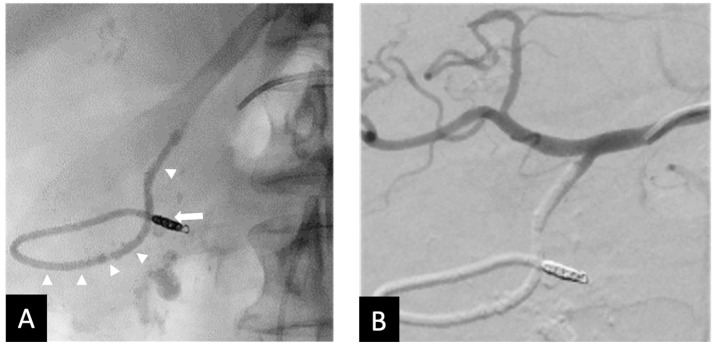
Seventy-three y.o. male with a large bleeding duodenal ulcer. Representative example of open-sandwich technique—(**A**) Post-embolization spot radiograph showing a 3 × 12 mm EmboldTM fibered coil (arrow) in the proximal right gastroepiploic artery (RGEA) and a continuous column of Ta-OCE (arrowhead) extending from the coil pack in the proximal RGEA to the proximal gastroduodenal artery (GDA) (**B**) Digital subtraction angiography (DSA) with contrast injection from the common hepatic artery (CHA) shows no flow in the GDA, RGEA, or any proximal branches, such as the supraduodenal artery (SDA). Image obtained from patient’s chart. Source: NK.

**Figure 4:**
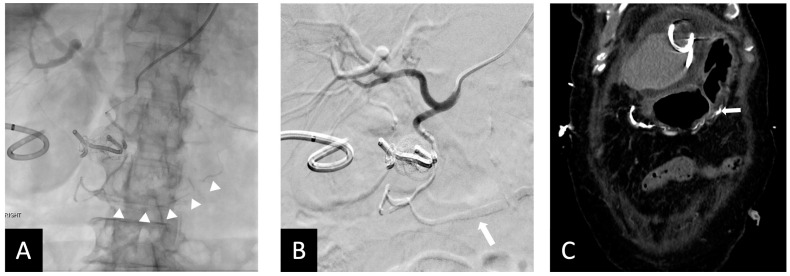
Fifty-five y.o male with bleeding duodenal ulcer. (**A**) Ta-OCE embolization of the GDA without using a backstop coil in distal GDA/proximal RGEA. (Arrow shows extension of Ta-OCE in the mid- to distal RGEA). (**B**) Post-GDA embolization DSA, demonstrating adequate GDA embolization and better visualization of Ta-OCE cast in the mid- and distal RGAE (arrow). (**C**) CT scan showing Ta-OCE extension into the distal RGEA post-embolization (arrow). Bleeding was successfully treated in this patient with no sign of bowel ischemia or injury. Image obtained from patient’s chart. Source: NK.

All 10 procedures were technically successful, where complete stasis was observed on angiography. Eight patients achieved clinical success and two experienced rebleeding within 24 hrs, as indicated by positive angiographic findings (contrast extravasation). No significant adverse events were observed. However, in one case where the 1.7 mm right gastroepiploic artery (RGEA) was not embolized, Ta-OCE migrated distally through the RGEA. Although no ischemic complications occurred, we recommend using a backstop coil to ensure precise and targeted embolization of the gastroduodenal artery (GDA) alone (Figure 2 and Figure 3). If the proximal RGEA is not embolized in vessels < 3 mm, Ta-OCE can extend into the mid- to distal RGAE, which is often clinically benign (Figure 4). However, in our experience, creating a backstop with a single coil in the proximal RGEA or distal GDA makes the GDA embolization more controlled.

**Figure 5:**
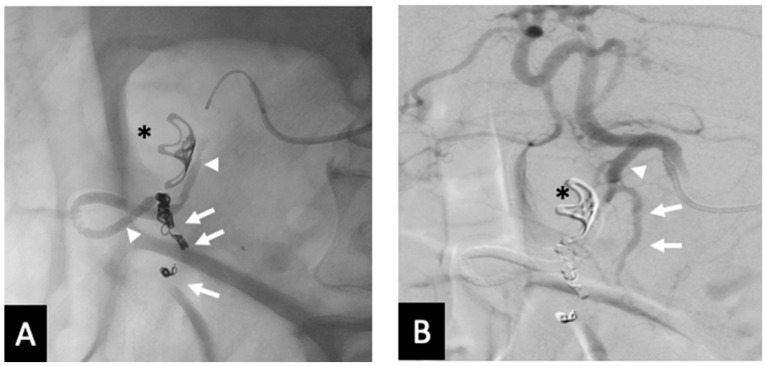
Rebleed example of unembolized SDA—(**A**) Post-embolization radiograph showing 3 mm coils in the proximal RGEA and inferior pancreaticoduodenal artery (arrows). Continuous column of Ta-OCE extending from the coil pack in the proximal RGEA to the proximal GDA (arrow head). An endoluminal clip placed during endoscopy is also demonstrated (*). (**B**) DSA with contrast injection from the CHA shows patent SDA (arrow) and proximal GDA (arrowhead). This patient required a second embolization to address the SDA. Image obtained from patient’s chart. Source: NK.

**Figure 6:**
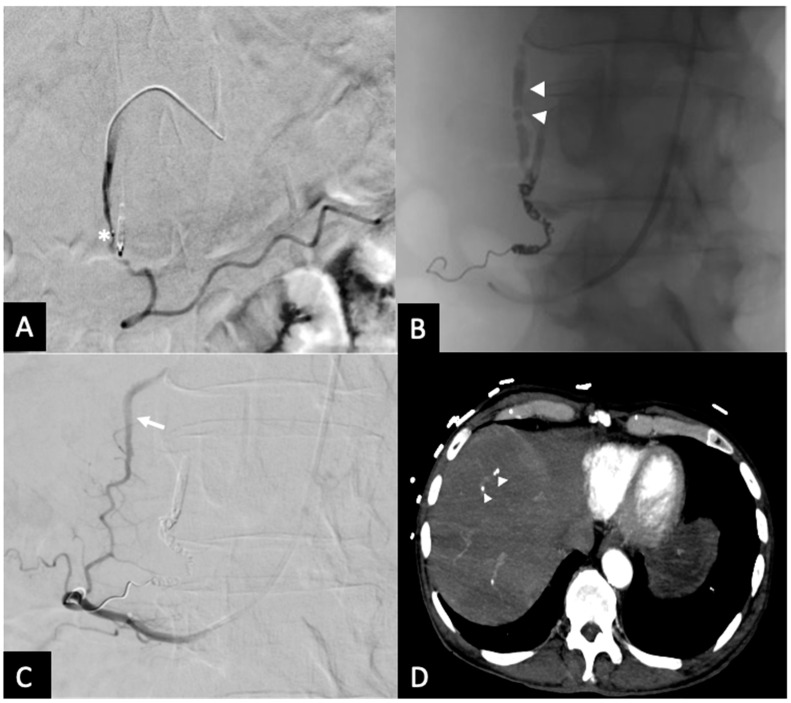
Rebleed example of unembolized RGEA—(**A**) Pre-embolization radiograph demonstrating the GDA and endoluminal clip placed by during endoscopy (*). (**B**) A single coil was placed in distal GDA, with Ta-OCE extending to proximal GDA (arrowheads). (**C**) Repeat angiography from the SMA approach upon the patient’s continued UGI bleeding demonstrated patent collateral flow from the posterior pancreaticoduodenal arcade (arrow). (**D**) Follow-up CT obtained between the 2 embolization procedures demonstrating separation of Ta-OCE (arrowhead) and embolization of branch hepatic arteries. Image obtained from patient’s chart. Source: NK.

In the two clinical failures, inadequate embolization was performed. Specifically, the GDA was not occluded proximally to its origin in one patient, leaving the SPDA patent, which led to rebleeding which was subsequently embolized with coils (Figure 5). The second patient required a second embolization to address collateral flow from the pancreatico duodenal arcade (Figure 6). This was thought to be due to inadequate distal placement of the backstop coil into the proximal RGEA. It is also worth noting that in one patient small fragments of Ta-OCE entered the hepatic artery due to rapid microcatheter movement with no clinical adverse events observed (Figure 6B). Of note, no ischemic bowel was noted. At 6 months, 9 of 10 patients did not require additional embolization, indicating durable clinical outcomes. One patient died due to complications from an invasive tumor, unrelated to the embolization procedure.

## 4. Discussion

UGIB is a common medical emergency and is a significant public health concern associated with high morbidity. The annual incidence of UGIB is approximately 80 to 150 per 100,000 population [1,2,5]. The mortality rate for UGIB ranges from 8 to 10% in de novo hospitalizations for bleeding and significantly increases when it occurs during hospitalization for another serious illness, with a 4-fold rise in mortality risk [3,5,23]. The situation becomes even more critical with recurrent bleeding, where mortality increases 10-fold, underscoring its grim outcome for patients [10,23]. The peptic ulcer is the most common underlying etiology for UGIB [3,16]. Timely management of UGIB correlates with favorable outcomes, which include early resuscitation followed by endoscopic interventions [3,6,7]. However, in a subset of patients for whom endoscopic treatment has failed or is contraindicated, such as by a high risk of rebleeding or a surgical history that precludes endoscopic access to the distal stomach and duodenum, TAE has emerged as a suitable alternative with a high success rate and lower adverse effects [9,10,12]. TAE can be performed through radial and femoral access. In our study, the choice of access was dependent on operator experience and patient anatomy. Reports have indicated that operator preference is a significant determining factor when opting for radial access [24]. However, patients displaying a Barbeau waveform D—indicative of inadequate ulnar–palmar arch patency—are not candidates for radial access [24]. TAE has reported technical success rates ranging from 69% to 100% and clinical success rates of 63% to 97% [7,8,14].

As outlined above, the sandwich technique is widely used when performing embolization for UGIB, with a reported 100% technical success rate and 86.6% clinical success rate [11]. The “sandwich” technique involves three main elements. First, retrograde sources of blood flow, including the right gastroepiploic artery (RGEA) and the pancreaticoduodenal arteries (PDA), are embolized with coils or plugs [5]. This is followed by gel foam slurry instillation within the GDA until stasis is achieved angiographically. Finally, the main trunk, and specifically the proximal aspect/inflow of the GDA, is embolized with coils or plugs, forming the “sandwich” [12]. This method prevents retrograde bleeding from collateral sources, such as the pancreaticoduodenal arcade fed by superior mesenteric artery (SMA) circulation.

While previous techniques and embolics have shown high success rates, it is vital for interventional radiologists to understand the different products, situations in which they can be used, and techniques that optimize their associated outcomes. During our initial experience using Ta-OCE with the proposed approach, 10 patients with UGIB who failed endoscopic treatment underwent GDA embolization using the “open sandwich” technique with Ta-OCE and a single distal coil. Notably, this approach, the “open-sandwich” technique, precludes the need for a terminal/proximal coil, simplifying the procedure and potentially reducing the risk of ischemic complication and additional bleeding following embolization [10]. Key factors could account for the successful implementation of this technique, such as careful consideration of injection speed and catheter size, both of which play a critical role in the delivery dynamics of Ta-OCE. For example, slower administration through non-high-flow microcatheters (2.0–2.4 Fr) facilitates more distal travel of Ta-OCE due to its shearing effect. However, breakage of the Ta-OCE stream can occur if the microcatheter is moved back too rapidly during continuous injection, posing a risk of embolization and migration of separated portions of the Ta-OCE column (Figure 6B). Despite its versatility and compatibility with a wide range of catheters, the high cost of Ta-OCE may limit its widespread use. Nevertheless, for operators that prefer using multiple detachable coils for GDA embolization, a single detachable coil and Ta-OCE can be more time- and cost-efficient.

Limited data exist on the use of this embolic agent. A few retrospective studies have reported on the use of Ta-OCE for GDA embolization [19,20,21]. In a study by Pal et al., 2 out of 11 patients underwent GDA embolization using Ta-OCE, and 100% technical and clinical success was reported [19]. Another single-center study evaluated Ta-OCE for various bleeding indications, reporting technical and clinical success rates of 97% and 93%, respectively, with four GDA embolizations performed out of 39 total vessels [21]. Of the four GDA embolizations reported, one patient experienced a Grade 5 adverse event due to an underlying complex medical condition [21]. Additionally, techniques like the ‘Aliquot’ method, where the agent is partially loaded into the microcatheter and then flushed, thus allowing the embolic to behave as a liquid, has been associated with adverse events [19,25,26]. In a recent case, embolic migration resulted in hepatic hypoperfusion and gallbladder ischemia, as well as infarction and necrosis of the anterior pancreatic head, neck, and body [26]. Currently, the FDA advises against this approach in lower GI bleeds as it can potentially lead to ischemia due to the embolization of multiple vasa recta [25,26]. Another technique involves manipulating injection speed, as mentioned earlier, through a 2.8 Fr microcatheter to create a ‘plug’, which could theoretically function as a coil alternative. However, the authors have not tested this method, and its effectiveness remains uncertain. Several authors have noted certain advantages of Ta-OCE, including no need for preparation, avoidance of catheter tip adhesion after delivery, and reduced CT streak artifact [19,21].

The sandwich technique, as mentioned earlier, utilizes gel foam. Gel foam offers several advantages, including cost-effectiveness, wide availability, and control over its consistency [12]. However, its use requires preparation and presents challenges in placement [19,20]. Recanalization is unpredictable, and gel foam particles’ of heterogeneous sizes, particularly the small ones, can lead to distal embolization into nearby collaterals or even reflux into non-target arteries [19,20]. The improper placement or very thin consistency of gel foam can increase the risk of complications, such as bowel ischemia [6,14]. Despite these challenges, gel foam remains a valuable option for embolization due to its affordability and accessibility [6,14].

Several studies have shown similar results with other embolics [4]. In a cohort of 75 patients with UGIB, arterial embolization was performed using metallic coils, polyvinyl alcohol particles (size range, 355–710 microns), gelatin sponge, and tissue adhesive. The authors reported a 98.7% technical success rate and 76% primary clinical success. It is worth noting that the authors concluded that coil-only embolization is a strong predictor of early rebleeding [27]. Similarly, in a cohort of 107 patients with cancer-related UGIB, various combinations of embolics were used—including microcoils with gelatin sponge particles and/or PVA particles, microcoils with NBCA, and gelatin sponge with PVA particles—while some patients received a single embolic agent [28]. This study reported a 99.1% technical success rate, a 56.1% clinical success rate, and a 30-day bleeding-related mortality rate of 13.1%. Although the embolic agent itself was not identified as a predictor of clinical success, the lack of heterogeneity in embolics and their combinations makes it difficult to draw definitive conclusions [28].

The use of multiple embolics congruently is a noteworthy concept within interventional radiology, as it allows interventionalists to leverage the synergistic features of different embolics. The results described indicate that Ta-OCE could be used in conjunction with other embolization agents, such as coils, to produce successful embolization of >3 mm vessels, such as splenic or internal iliac arteries, more efficiently compared to coil embolization alone. This is consistent when other embolics are used with coils. In a study of 30 patients with massive bleeding from gastric or duodenal ulcers, TAE using N-butyl cyanoacrylate NBCA (with lipiodol, sometimes combined with coils/gel foam) achieved complete hemostasis in 85.7% of cases versus 78.3% without a secondary embolic agent [29].

Future research should focus on prospective, multicenter studies with larger sample sizes and longer follow-up periods to identify any late-onset complications. Moreover, ongoing research, such as the OCCLUDE registry, an open-label, single-arm, multicenter US registry of patients who will undergo embolization with Ta-OCE, will further provide more robust insight into its effectiveness and safety as an embolic [30]. This multicenter study will further validate our preliminary findings and establish more comprehensive clinical guidelines for UGIB management using Ta-OCE.

This study has several limitations. First, the small sample size and retrospective design reduce statistical power, increasing the risk of a type II error and introducing selection and reporting biases that limit the generalizability of our findings. This retrospective approach and limited sample size were primarily due to constraints in available data and the given novelty of this product. Additionally, we did not control for confounding factors such as gender, smoking, alcohol intake, and drug use, all of which may affect UGIB severity [31]. Our study population might not fully represent the broader patient population with UGIB, potentially affecting the observed outcomes. Only patients with UGI bleeding requiring GDA embolization were included in this analysis; therefore, the utility of our technique for treating UGI bleeding involving embolization of other arteries cannot be evaluated. Moreover, conclusions regarding the effectiveness of Ta-OCE compared with other embolic agents cannot be drawn. These results should be interpreted as preliminary, and further prospective, methodologically robust clinical trials are warranted to validate our findings.

## 5. Conclusions

In conclusion, Ta-OCE demonstrated 100% technical success and durable treatment effectiveness in patients. Our initial experience of GDA embolization with Ta-OCE is feasible, safe, and effective. Additionally, Ta-OCE can be effectively combined with other embolization agents to achieve successful embolization. A larger multicentered prospective clinical trial with an extended follow-up period is warranted to validate these results.

## Figures and Tables

**Table 1:** Patient characteristics.

Characteristics		*n* = 10 (%)
Age		67.3 ± 15.2
Sex	Male	8 (80%)
	Female	2 (20%)
Underlying Bleeding Cause	Duodenal/Gastric Ulcer	6
	Complication related to endoscopic placement of transduodenal stent to drain the gallbladder	1
	Tumor invasion of duodenum	2
	Peripancreatic hemorrhage	1
Hemodynamic Status *	Stable	6 (60%)
	Unstable	4 (40%)
Indication	Prophylactic	6 (60%)
	Active	4 (40%)

* Hemodynamically unstable (heart rate, >100 beats/min; hypotension [systolic blood pressure, <90 mm Hg]) or on pressors.

## Data Availability

The datasets used and/or analyzed during the current study are available from the corresponding author on reasonable request.
